# Supplementary material for: Analysis of Circulating Immune Subsets in Primary Colorectal Cancer
Source: Cancers (Basel). 2022 Dec 12;14(24):6105. doi: 10.3390/cancers14246105 (PMC9776578; doi:10.3390/cancers14246105)
Supplement: Supplementary file 1 [file cancers-14-06105-s001.zip › Table S1.pdf]

Table S1. Flow cytometry panels applied to identify peripheral blood B-, T-, and Innate immune subsets

Marker	Fluorochrome	Clone	Catalog number	Source
**Flow panel 1 B lymphocytes subsets**				
IgM	BV510	G20-127	563113	BD Biosciences
CD38	BV605	HB7	562665	BD Biosciences
CD45	BV650	HI30	563717	BD Biosciences
CD27	BV786	L128	563327	BD Biosciences
CD19	FITC	HIB19	555412	BD Biosciences
CD3	PerCP Cy5.5	UCHT1	300430	BioLegend
CD10	PE	HI10a	555375	BD Biosciences
CD24	PE-CF594	ML5	562405	BD Biosciences
IgD	PE-Cy7	IA6-2	561314	BD Biosciences
CD20*	APC-H7	H1	561172	BD Biosciences
**Flow panel 2 T lymphocytes subsets**				
CD4	BUV395	SK3	563550	BD Biosciences
CD197	BV421	150503	562555	BD Biosciences
CD194	BV510	1G1	563066	BD Biosciences
CD38	BV605	HB7	562665	BD Biosciences
CD45	BV650	HI30	563717	BD Biosciences
CD25	BB515	2A3	564467	BD Biosciences
CD3	PerCP Cy5.5	UCHT1	300430	BioLegend
CD196	PE	11A9	559562	BD Biosciences
CD127	PE-CF594	HIL-7R-M21	562397	BD Biosciences
CD45RO	PE-Cy7	UCHL1	560608	BD Biosciences
HLA-DR	APC	G46-6	559866	BD Biosciences
CD8	APC-H7	SK1	560179	BD Biosciences
**Flow panel 3 Innate Immune subsets**				

CD69	BUV395	FN50	564364	BD Biosciences
HLA-DR	BV421	G46-6	562804	BD Biosciences
CD14	BV510	MφP9	563079	BD Biosciences
CD45	BV650	HI30	563717	BD Biosciences
CD33	BV786	WM53	740974	BD Biosciences
CD16	FITC	B73.1	561308	BD Biosciences
CD3	PerCP Cy5.5	UCHT1	300430	BioLegend
CD11c	PE	B-ly6	555392	BD Biosciences
CD15	PE-CF594	W6D3	562372	BD Biosciences
CD11b	PE-Cy7	ICRF44	557743	BD Biosciences
CD66b	AF647	G10F5	561645	BD Biosciences
CD56	APC-R700	NCAM16.2	565139	BD Biosciences
CD8	APC-H7	SK1	560179	BD Biosciences

*, represent the intracellular staining antibody.

Abbreviations: AF, Alexa fluor; APC, allophycocyanin; BV, brilliant violet; FITC, fluorescein isothiocyanate; PE, phycoerythrin; PE-Cy7, phycoerythrin-cyanine7; PerCP, peridinin chlorophyll protein complex; V500, violet500. BUV, brilliant ultraviolet.
