# Supplementary material for: Analysis of Circulating Immune Subsets in Primary Colorectal Cancer
Source: Cancers (Basel). 2022 Dec 12;14(24):6105. doi: 10.3390/cancers14246105 (PMC9776578; doi:10.3390/cancers14246105)
Supplement: Supplementary file 1 [file cancers-14-06105-s001.zip › Table S2.pdf]

Table S2. Immunophenotyping of B lymphocytes, T lymphocytes, and Innate immune subsets

Immune cells	Immunophenotyping
**B lymphocytes subsets**	
B lymphocytes	$CD45^{+}CD3^{-}CD19^{+}$
Pre-B cells	$CD45^{+}CD3^{-}CD19^{+}CD10^{+}IgM^{-}CD24^{high}CD38^{high}$ $CD20^{+}$
Transitional B cells	$CD45^{+}CD3^{-}CD19^{+}CD24^{high}CD38^{high}CD10^{+}$ $CD27^{-}$
Breg-Immature cells	$CD45^{+}CD3^{-}CD19^{+}CD24^{high}CD38^{high}CD27^{-}$
Breg-B10 cells	$CD45^{+}CD3^{-}CD19^{+}CD24^{high}CD27^{+}$
Naïve B cells	$CD45^{+}CD3^{-}CD19^{+}CD27^{-}IgD^{+}$
Non-Classic switched memory B cells	$CD45^{+}CD3^{-}CD19^{+}CD27^{+}IgD^{+}$
Class switched memory B cells	$CD45^{+}CD3^{-}CD19^{+}CD27^{+}IgD^{-}$
Plasmablasts	$CD45^{+}CD3^{-}CD19^{+}CD27^{+}CD20^{low/-}CD38^{high}$
**T lymphocytes subsets**	
T lymphocytes	$CD45^{+}CD3^{+}$
CD8T cells	$CD45^{+}CD3^{+}CD8^{+}$
Activated CD8T cells	$CD45^{+}CD3^{+}CD8^{+}CD38^{+}HLA^{-}DR^{+}$
Effector CD8T cells	$CD45^{+}CD3^{+}CD8^{+}CD197^{-}CD45RO^{-}$
Effector memory CD8T cells	$CD45^{+}CD3^{+}CD8^{+}CD197^{-}CD45RO^{+}$
Naïve CD8T cells	$CD45^{+}CD3^{+}CD8^{+}CD197^{+}CD45RO^{-}$
Central memory CD8T cells	$CD45^{+}CD3^{+}CD8^{+}CD197^{+}CD45RO^{+}$
Th cells	$CD45^{+}CD3^{+}CD4^{+}$
Activated Th cells	$CD45^{+}CD3^{+}CD4^{+}CD38^{+}HLA\text{-}DR^{+}$
Effector Th cells	$CD45^{+}CD3^{+}CD4^{+}CD197^{-}CD45RO^{-}$
Effector memory Th cells	$CD45^{+}CD3^{+}CD4^{+}CD197^{-}CD45RO^{+}$
Naïve Th cells	$CD45^{+}CD3^{+}CD4^{+}CD197^{+}CD45RO^{-}$
Central memory Th cells	$CD45^{+}CD3^{+}CD4^{+}CD197^{+}CD45RO^{+}$
Th17 cells	$CD45^{+}CD3^{+}CD4^{+}CD194^{+}CD196^{+}$

Th1 cells	$CD45^{+}CD3^{+}CD4^{+}CD194^{-}CD196^{-}$
Th2 cells	$CD45^{+}CD3^{+}CD4^{+}CD194^{+}CD196^{-}$
Treg cells	$CD45^{+}CD3^{+}CD4^{+}CD25^{high}CD127^{low}$
Memory Treg cells	$CD45^{+}CD3^{+}CD4^{+}CD25^{high}CD127^{low}CD194^{+}$ $CD45RO^{+}$
Naive Treg cells	$CD45^{+}CD3^{+}CD4^{+}CD25^{high}CD127^{low}CD194^{+}$ $CD45RO^{-}$
Activated Treg cells	$CD45^{+}CD3^{+}CD4^{+}CD25^{high}CD127^{low}CD194^{+}$ $HLA\text{-}DR^{+}$
**Innate immune subsets**	
Neutrophils	$CD45^{+}CD14^{-}CD66b^{+}CD15^{+}CD11b^{+}CD16^{+/-}$
Classical Monocytes	$CD45^{+}CD66b^{-}HLA\text{-}DR^{+}CD14^{high}$ $CD16^{-}$
Non-Classical Monocytes	$CD45^{+}CD66b^{-}HLA\text{-}DR^{low/+}CD14^{low/+}$ $CD16^{+}$
Intermediate Monocytes	$CD45^{+}CD66b^{-}HLA\text{-}DR^{high}CD14^{high}$ $CD16^{+}$
Dendritic cells	$CD45^{+}CD66b^{-}HLA\text{-}DR^{+}CD14^{-/low}$ $CD16^{-}$
MDSCs	$CD45^{+}CD33^{+}$ $HLA\text{-}DR^{-/low}$ $CD11b^{+}$
PMN-MDSCs	$CD45^{+}CD33^{+}$ $HLA\text{-}DR^{-/low}$ $CD11b^{+}CD14^{-}CD15^{+}$
M-MDSCs	$CD45^{+}CD33^{+}$ $HLA\text{-}DR^{-/low}$ $CD11b^{+}CD14^{+}CD15^{-}$
Early stage-MDSCs	$CD45^{+}CD33^{+}$ $HLA\text{-}DR^{-/low}$ $CD11b^{+}CD14^{-}$ $CD15^{-}CD56^{-}$
NK cells	$CD45^{+}CD14^{-}CD3^{-}CD56^{+}$
$CD56^{bright}$ NK cells	$CD45^{+}CD14^{-}CD3^{-}CD56^{high}$
$CD8^{+}CD56^{bright}$ NK cells	$CD45^{+}CD14^{-}CD3^{-}CD56^{high}CD8^{+}$
$CD16^{+}CD56^{bright}$ NK cells	$CD45^{+}CD14^{-}CD3^{-}CD56^{high}CD16^{+}$
$CD69^{+}CD56^{bright}$ NK cells	$CD45^{+}CD14^{-}CD3^{-}CD56^{high}CD69^{+}$
$CD56^{dim}$ NK cells	$CD45^{+}CD14^{-}CD3^{-}CD56^{+/Low}$
$CD8^{+}CD56^{dim}$ NK cells	$CD45^{+}CD14^{-}CD3^{-}CD56^{dim}CD8^{+}$
$CD16^{+}CD56^{dim}$ NK cells	$CD45^{+}CD14^{-}CD3^{-}CD56^{dim}CD16^{+}$
$CD69^{+}CD56^{dim}$ NK cells	$CD45^{+}CD14^{-}CD3^{-}CD56^{dim}CD69^{+}$
NKT cells	$CD45^{+}CD14^{-}CD3^{+}CD56^{+}$
$CD8^{+}$NKT cells	$CD45^{+}CD14^{-}CD3^{+}CD56^{+}CD8^{+}$
$CD16^{+}$NKT cells	$CD45^{+}CD14^{-}CD3^{+}CD56^{+}CD16^{+}$

$CD69^+$NKT cells	$CD45^+CD14^-CD3^+CD56^+CD69^+$

Abbreviations: Breg, regulatory B; Th, T helper; Treg, regulatory T; MDSCs, myeloid-derived suppressor cells; PMN-MDSCs, polymorphonuclear MDSCs; M-MDSCs, Monocytic MDSCs; NK, Natural killer; NKT, Natural killer T.
