# Supplementary material for: Analysis of Circulating Immune Subsets in Primary Colorectal Cancer
Source: Cancers (Basel). 2022 Dec 12;14(24):6105. doi: 10.3390/cancers14246105 (PMC9776578; doi:10.3390/cancers14246105)
Supplement: Supplementary file 1 [file cancers-14-06105-s001.zip › Table S3.pdf]

Table S3. Comparison of circulating immune subsets between CRC patients and healthy controls

Immune cells	Healthy controls (N = 11)	CRC patients (N = 12)	Corrected *P*-value[#]
B % Leukocytes	3.10 ± 1.21	1.72 ± 1.43	**0.0421**
Pre % B	0.53 ± 0.34	1.39 ± 1.70	0.5772
Transitional % B	1.28 ± 0.70	3.43 ± 3.61	0.2850
Plasmablast % B	1.16 ± 1.17	5.31 ± 10.52	0.0503
NCSM % B	9.14 ± 5.63	8.73 ± 6.28	0.8650
CSM % B	16.84 ± 13.02	16.20 ± 9.96	0.9509
Naive % B	59.95 ± 20.35	53.50 ± 21.90	0.6556
Breg-B10 % B	20.63 ± 12.38	12.09 ± 8.09	0.2326
Breg-Immature % B	4.24 ± 1.89	5.54 ± 5.03	0.9889
T % Leukocytes	29.15 ± 4.96	18.10 ± 8.03	**0.0184**
CD8T % Leukocytes	8.76 ± 3.93	5.45 ± 3.44	0.1391
Activated % CD8T	2.23 ± 0.96	5.84 ± 2.97	**0.0107**
Naive % CD8T	16.79 ± 14.84	15.59 ± 7.17	0.7963
EM % CD8T	32.96 ± 17.28	36.54 ± 12.93	0.6556
E % CD8T	44.32 ± 20.54	39.18 ± 17.30	0.5692
CM % CD8T	5.92 ± 3.82	8.69 ± 4.57	0.3331
Th % Leukocytes	18.84 ± 4.43	11.13 ± 5.17	**0.0243**
Activated % Th	1.40 ± 0.87	3.00 ± 1.08	**0.0107**
Naive % Th	7.87 ± 6.43	30.04 ± 14.88	**0.0088**
EM % Th	51.07 ± 15.45	33.67 ± 15.60	0.0507
E % Th	35.16 ± 15.95	8.20 ± 4.13	**0.0088**
CM % Th	5.92 ± 6.46	28.11 ± 14.64	**0.0088**
Th1 % Th	62.77 ± 10.55	64.51 ± 13.81	0.4574
Th2 % Th	8.71 ± 3.59	10.03 ± 6.68	1.0000
Th17 % Th	10.37 ± 5.53	10.95 ± 8.74	0.9831

Tregs % Th	6.94 ± 3.17	8.91 ± 3.95	0.4574
Naive % Tregs	6.47 ± 7.71	1.70 ± 1.47	0.2140
Memory % Tregs	41.36 ± 20.09	58.28 ± 14.31	0.2140
Activated % Tregs	14.30 ± 6.66	12.83 ± 8.30	0.6118
Non-Classical Monocyte % Leukocytes	0.60 ± 0.26	0.24 ± 0.15	**0.0125**
Intermediate-Monocyte % Leukocytes	0.04 ± 0.02	0.06 ± 0.09	0.6621
Classical-Monocyte % Leukocytes	2.24 ± 1.95	1.91 ± 1.37	0.9946
Total Monocyte % Leukocytes	2.93 ± 1.95	2.21 ± 1.46	0.7087
Neutrophil % Leukocytes	54.16 ± 7.93	61.41 ± 14.75	0.5359
DC % Leukocytes	2.93 ± 2.82	0.59 ± 0.69	**0.0265**
MDSC % Leukocytes	0.37 ± 0.27	0.28 ± 0.16	0.7266
PMN-MDSC % MDSC	26.04 ± 22.18	68.27 ± 12.84	**0.0107**
M-MDSC % MDSC	38.53 ± 27.40	17.11 ± 12.60	0.2065
E-MDSC % MDSC	24.00 ± 14.37	11.29 ± 7.91	0.1096
NK % Leukocytes	3.38 ± 1.85	4.09 ± 4.46	0.8650
$CD56^{dim}$ % NK	92.64 ± 4.89	90.08 ± 5.39	0.2787
$CD69^{+}$ % $CD56^{dim}$NK	12.93 ± 27.45	19.39 ± 25.30	0.0662
CD69 ($CD56^{dim}$ NK) *	279.00 (269.50, 297.00)	116.5 (105.58, 201.00)	**0.0277**
$CD16^{+}$ % $CD56^{dim}$NK	93.96 ± 4.74	80.19 ± 21.89	0.2004
CD16 ($CD56^{dim}$ NK) *	480.00 (394.50, 585.50)	284.00 (145.00, 591.50)	0.4222
$CD8^{+}$ % $CD56^{dim}$NK	30.59 ± 18.38	32.61 ± 15.86	0.7266
CD8 ($CD56^{dim}$ NK) *	1345.00 (1302.00, 1712.00)	1482.50 (1226.75, 1827.25)	0.9264

CD56^{bright} % NK	7.35 ± 4.89	9.86 ± 5.22	0.2912
CD69^{+} % CD56^{bright}NK	14.35 ± 27.99	15.15 ± 25.91	0.4574
CD69 (CD56^{bright} NK) *	245.00 (223.50, 296.50)	112.00 (76.38, 145.00)	**0.0277**
CD16^{+} % CD56^{bright}NK	40.71 ± 13.95	29.48 ± 21.38	0.2737
CD16 (CD56^{bright} NK) *	255.00 (137.00, 304.00)	150.00 (90.70, 256.25)	0.4180
CD8^{+} % CD56^{bright}NK	24.70 ± 22.80	21.66 ± 10.74	0.7087
CD8 (CD56^{bright} NK) *	1601.00 (1129.5, 1775.00)	1441.50 (1346.50, 1586.00)	0.8795
NKT % Leukocytes	2.27 ± 2.54	1.37 ± 1.35	0.2065
CD69^{+} % NKT	14.28 ± 27.71	20.19 ± 24.09	0.1002
CD69 (NKT) *	303.00 (285.50, 352.50)	178.00 (152.75, 356.75)	0.1815
CD16^{+} % NKT	6.48 ± 7.15	5.51 ± 4.32	0.7877
CD16 (NKT) *	97.70 (85.15, 110.00)	86.45 (72.33, 131.50)	0.8795
CD8^{+} % NKT	67.57 ± 19.76	55.18 ± 12.64	0.2737
CD8 (NKT) *	5726.00 (4821.00, 7183.00)	7563.00 (5321.25, 8728.75)	0.4222

*, represent the MFI value expressed as the median with 95% confidence interval. All other data were presented with the Mean ± SD (%). The Benjamini-Hochberg method was used to correct *P*-value for multiple testing, which was indicated by *P*-value$^{\#}$. *P*-value$^{\#}$ < 0.05 was regarded as statistically different.

Abbreviation: NCSM: non-class switched memory; CSM: class switched memory; Breg, regulatory B; EM, effector memory; E, effector; CM, central memory; Treg, regulatory T cells; NK, natural killer; NKT, natural killer T; DC, dendritic cell; PMN-MDSC, polymorphonuclear MDSC; M-MDSC, mononuclear MDSC; E-MDSC, early-stage MDSC.
