# Supplementary material for: Analysis of Circulating Immune Subsets in Primary Colorectal Cancer
Source: Cancers (Basel). 2022 Dec 12;14(24):6105. doi: 10.3390/cancers14246105 (PMC9776578; doi:10.3390/cancers14246105)
Supplement: Supplementary file 1 [file cancers-14-06105-s001.zip › Table S4.pdf]

Table S4. Identification of circulating immune subsets to differentiate CRC patients from healthy controls through univariable logistic regression

Immune cells	*P* value
B_Leucocytes	0.053
Pre_B	0.23
Transitional_B	0.179
Plasmablast_B	0.187
NCSM_B	0.869
CSM_B	0.894
Naive_B	0.475
Breg_B10_B	0.086
Breg_Immature_B	0.438
T_Leucocytes	**0.021**
CD8T_Leucocytes	0.076
Activated_CD8T	**0.021**
Naive_CD8T	0.803
EM_CD8T	0.579
E_CD8T	0.522
CM_CD8T	0.152
Th_Leucocytes	**0.012**
Activated_Th	**0.018**
Naive_Th	**0.017**
EM_Th	**0.034**
E_Th	**0.037**
CM_Th	**0.007**
Th1_Th	0.739
Th2_Th	0.567
Th17_Th	0.853
Tregs_Th	0.221
Naive_Tregs	0.062
Memory_Tregs	0.066
Activated_Tregs	0.645
Non-Classical Monocyte_Leucocytes	**0.038**
Intermediate Monocyte_Leucocytes	0.453
Classical Monocyte_Leucocytes	0.639
Total Monocyte_Leucocytes	0.328
Neutrophil_Leucocytes	0.184
NK_Leucocytes	0.628
$CD56^{dim}$_NK	0.268
CD69_$CD56^{dim}$ NK	0.568
CD16_$CD56^{dim}$ NK	0.076
CD8_$CD56^{dim}$ NK	0.778
$CD56^{bright}$_NK	0.269

CD69_CD56^{bright} NK	0.944
CD16_CD56^{bright} NK	0.172
CD8_CD56^{bright} NK	0.681
NKT_Leucocytes	0.345
CD69_NKT	0.594
CD16_NKT	0.693
CD8_NKT	0.107
DC_Leucocytes	0.079
MDSC_Leucocytes	0.349
PMN.MDSC_MDSC	**0.006**
M.MDSC_MDSC	0.058
Early-stage MDSC	**0.037**

Note: Each immune subset was expressed as the percentage of source cells annotated following the underscore.

Abbreviation: NCSM: non-class switched memory; CSM: class switched memory; Breg, regulatory B cells; EM, effector memory; E, effector; CM, central memory; Tregs, regulatory T cells; NK, natural killer; NKT, natural killer T; DC, Dendritic cell; PMN.MDSC, polymorphonuclear MDSC; M.MDSC, mononuclear MDSC.
