# Supplementary material for: Analysis of Circulating Immune Subsets in Primary Colorectal Cancer
Source: Cancers (Basel). 2022 Dec 12;14(24):6105. doi: 10.3390/cancers14246105 (PMC9776578; doi:10.3390/cancers14246105)
Supplement: Supplementary file 1 [file cancers-14-06105-s001.zip › Table S5.pdf]

Table S5. The accuracy of individual immune subset or the combination of different immune subsets as the classifier to distinguish CRC patients from healthy controls by Support Vector Machine learning algorithm

Feature Combinations	10 × CV Accuracy
NC.Monocyte_Leukocytes	0.741
NC.Monocyte_Leukocytes and E_Th	0.863
NC.Monocyte_Leukocytes, E_Th, and T_Leukocytes	0.856
NC.Monocyte_Leukocytes, E_Th, T_Leukocytes, and Activated_Th	0.882
NC.Monocyte_Leukocytes, E_Th, T_Leukocytes, Activated_Th, and Activated_CD8T	0.863
NC.Monocyte_Leukocytes, E_Th, T_Leukocytes, Activated_Th, Activated_CD8T, and Th_Leukocytes	0.877
NC.Monocyte_Leukocytes, E_Th, T_Leukocytes, Activated_Th, Activated_CD8T, Th_Leukocytes, and Naïve_Th	0.936
NC.Monocyte_Leukocytes, E_Th, T_Leukocytes, Activated_Th, Activated_CD8T, Th_Leukocytes, Naïve_Th, and PMN.MDSC_MDSC	0.924
NC.Monocyte_Leukocytes, E_Th, T_Leukocytes, Activated_Th, Activated_CD8T, Th_Leukocytes, Naïve_Th, PMN.MDSC_MDSC, and EM_Th	0.917
NC.Monocyte_Leukocytes, E_Th, T_Leukocytes, Activated_Th, Activated_CD8T, Th_Leukocytes, Naïve_Th, PMN.MDSC_MDSC, EM_Th, and CM_Th	0.911
NC.Monocyte_Leukocytes, E_Th, T_Leukocytes, Activated_Th, Activated_CD8T, Th_Leukocytes, Naïve_Th, PMN.MDSC_MDSC, EM_Th, CM_Th, and E.MDSC_MDSC	0.924

Abbreviation: 10 × CV, ten-fold cross validation; NC.Monocyte_Leukocytes; non-classical monocyte (% of Leukocytes); E_Th, effector Th (% of Th); Activated_Th, activated Th (% of Th); T_Leukocytes, T (% of Leukocytes); Activated_CD8T, activated CD8T (% of CD8T); Th_Leukocytes, Th (% of Leukocytes); Naïve_Th, naïve

Th (% of Th); PMN.MDSC_MDSC, polymorphonuclear MDSC (% of MDSC); EM_Th, effector memory Th (% of Th); CM_Th, central memory Th (% of Th); E.MDSC_MDSC, early-stage MDSC (% of MDSC); MDSC, myeloid-derived suppressor cell; Th, T helper, CD8T, $CD8^{+}$ T.
