# Supplementary material for: Analysis of Circulating Immune Subsets in Primary Colorectal Cancer
Source: Cancers (Basel). 2022 Dec 12;14(24):6105. doi: 10.3390/cancers14246105 (PMC9776578; doi:10.3390/cancers14246105)
Supplement: Supplementary file 1 [file cancers-14-06105-s001.zip › Table S6.pdf]

Table S6. Correlation analysis between circulating Th cells and common DEGs in healthy controls

Genes	Immune cells	Correlation coefficient	*P*-value
GRINA	Th cells	-0.51	2.07E-05
PDCD4	Th cells	0.65	1.24E-08
MIER3	Th cells	0.65	9.88E-09
NR3C2	Th cells	0.81	8.88E-16
ABHD3	Th cells	0.21	1.09E-01
NAP1L2	Th cells	0.66	4.26E-09
P2RY14	Th cells	0.44	3.21E-04
GIMAP7	Th cells	0.73	1.25E-11
ACADM	Th cells	0.69	5.76E-10
PRKACB	Th cells	0.77	3.04E-13
MGAT4A	Th cells	0.69	4.99E-10
GPRASP1	Th cells	0.65	1.48E-08
NAP1L3	Th cells	0.69	4.09E-10
SYTL2	Th cells	0.37	2.83E-03
KLRB1	Th cells	0.60	2.17E-07
BEX4	Th cells	0.63	4.87E-08
KLRF1	Th cells	0.40	1.23E-03
MS4A1	Th cells	0.55	3.39E-06
SH2D1B	Th cells	0.11	4.02E-01
TGFBR3	Th cells	0.15	2.36E-01
CAMK4	Th cells	0.83	0.00E+00
FCRL3	Th cells	0.39	1.82E-03
SMCHD1	Th cells	-0.11	3.95E-01
CD96	Th cells	0.77	3.14E-13
P2RY10	Th cells	0.67	1.82E-09
ZNF304	Th cells	0.50	3.40E-05

RGS18	Th cells	0.13	2.96E-01
ABCA5	Th cells	0.55	3.06E-06
GZMA	Th cells	0.54	6.13E-06
CD52	Th cells	0.71	7.96E-11
ZNF831	Th cells	0.37	2.82E-03
GIMAP5	Th cells	0.32	1.22E-02
THEMIS	Th cells	0.73	1.39E-11
EVI2A	Th cells	0.52	1.26E-05
GPR183	Th cells	0.80	8.44E-15
TRAT1	Th cells	0.83	0.00E+00
ZNF439	Th cells	0.58	7.30E-07
CD69	Th cells	0.69	6.96E-10
GPR174	Th cells	0.71	1.22E-10

Abbreviation: Th cells, T helper cells.
