# Supplementary material for: Analysis of Circulating Immune Subsets in Primary Colorectal Cancer
Source: Cancers (Basel). 2022 Dec 12;14(24):6105. doi: 10.3390/cancers14246105 (PMC9776578; doi:10.3390/cancers14246105)
Supplement: Supplementary file 1 [file cancers-14-06105-s001.zip › Table S7.pdf]

Table S7. Correlation analysis between circulating Th cells and common DEGs in CRC patients

| Genes | Immune cells | Correlation coefficient | *P*-value |
|---|---|---|---|
| GRINA | Th cells | -0.64 | 8.78E-10 |
| PDCD4 | Th cells | 0.77 | 3.11E-15 |
| MIER3 | Th cells | 0.63 | 3.00E-09 |
| NR3C2 | Th cells | 0.87 | 0.00E+00 |
| ABHD3 | Th cells | 0.33 | 4.21E-03 |
| NAP1L2 | Th cells | 0.63 | 2.96E-09 |
| P2RY14 | Th cells | 0.39 | 5.93E-04 |
| GIMAP7 | Th cells | 0.84 | 0.00E+00 |
| ACADM | Th cells | 0.68 | 2.96E-11 |
| PRKACB | Th cells | 0.77 | 2.66E-15 |
| MGAT4A | Th cells | 0.61 | 8.06E-09 |
| GPRASP1 | Th cells | 0.72 | 5.09E-13 |
| NAP1L3 | Th cells | 0.76 | 8.88E-15 |
| SYTL2 | Th cells | 0.61 | 9.26E-09 |
| KLRB1 | Th cells | 0.67 | 1.27E-10 |
| BEX4 | Th cells | 0.73 | 3.44E-13 |
| KLRF1 | Th cells | 0.66 | 1.65E-10 |
| MS4A1 | Th cells | 0.64 | 7.71E-10 |
| SH2D1B | Th cells | 0.50 | 6.13E-06 |
| TGFBR3 | Th cells | 0.58 | 9.26E-08 |
| CAMK4 | Th cells | 0.85 | 0.00E+00 |
| FCRL3 | Th cells | 0.58 | 9.12E-08 |
| SMCHD1 | Th cells | 0.08 | 5.14E-01 |
| CD96 | Th cells | 0.82 | 0.00E+00 |
| P2RY10 | Th cells | 0.77 | 1.11E-15 |
| ZNF304 | Th cells | 0.68 | 2.42E-11 |

| | | | |
|---|---|---|---|
| RGS18 | Th cells | 0.18 | 1.32E-01 |
| ABCA5 | Th cells | 0.57 | 1.06E-07 |
| GZMA | Th cells | 0.63 | 2.16E-09 |
| CD52 | Th cells | 0.60 | 2.59E-08 |
| ZNF831 | Th cells | 0.70 | 3.95E-12 |
| GIMAP5 | Th cells | 0.54 | 9.13E-07 |
| THEMIS | Th cells | 0.80 | 0.00E+00 |
| EVI2A | Th cells | 0.32 | 5.11E-03 |
| GPR183 | Th cells | 0.88 | 0.00E+00 |
| TRAT1 | Th cells | 0.86 | 0.00E+00 |
| ZNF439 | Th cells | 0.68 | 3.25E-11 |
| CD69 | Th cells | 0.70 | 4.48E-12 |
| GPR174 | Th cells | 0.77 | 3.11E-15 |

Abbreviation: Th cells, T helper cells.
