# Supplementary figures and images for: Analysis of Circulating Immune Subsets in Primary Colorectal Cancer
Source: Cancers (Basel). 2022 Dec 12;14(24):6105. doi: 10.3390/cancers14246105 (PMC9776578; doi:10.3390/cancers14246105)

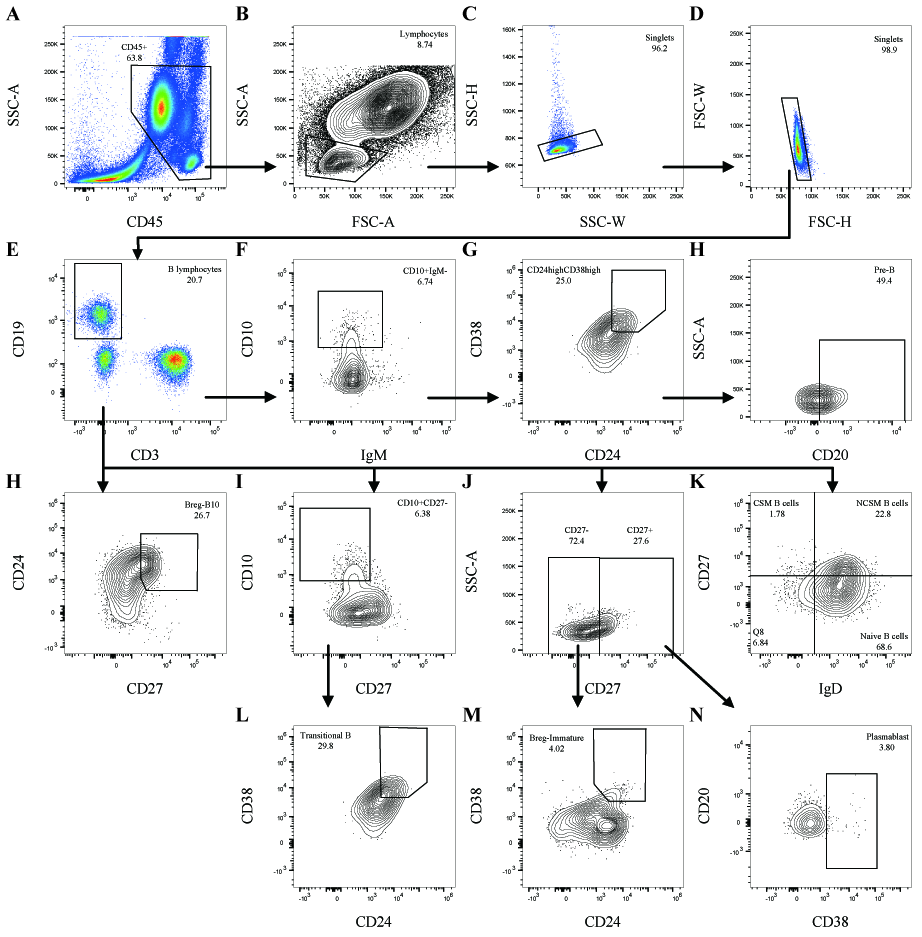

Supplement: Supplementary file 1 [file cancers-14-06105-s001.zip › Figure S1.tif]

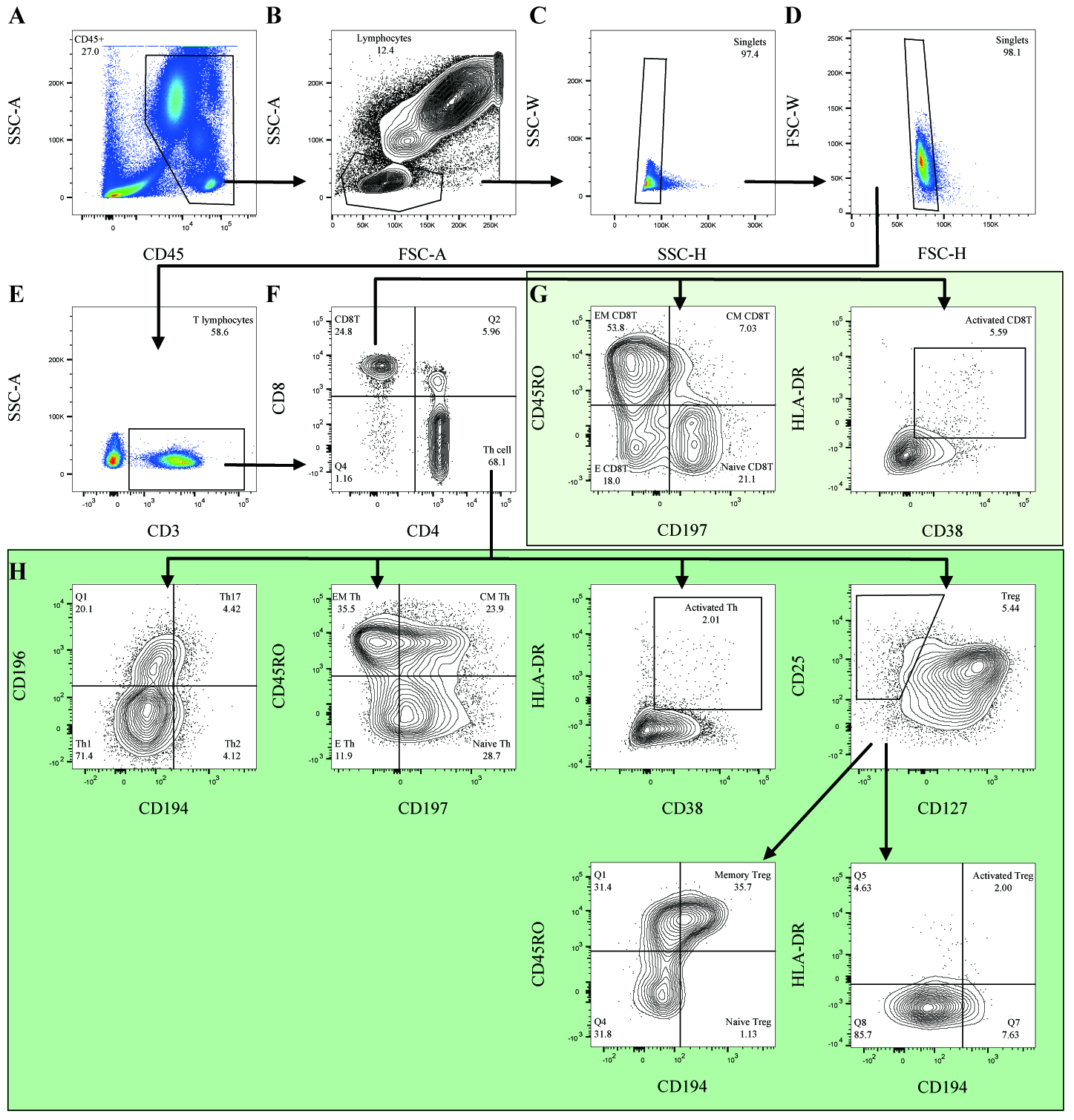

Supplement: Supplementary file 1 [file cancers-14-06105-s001.zip › Figure S2.tif]

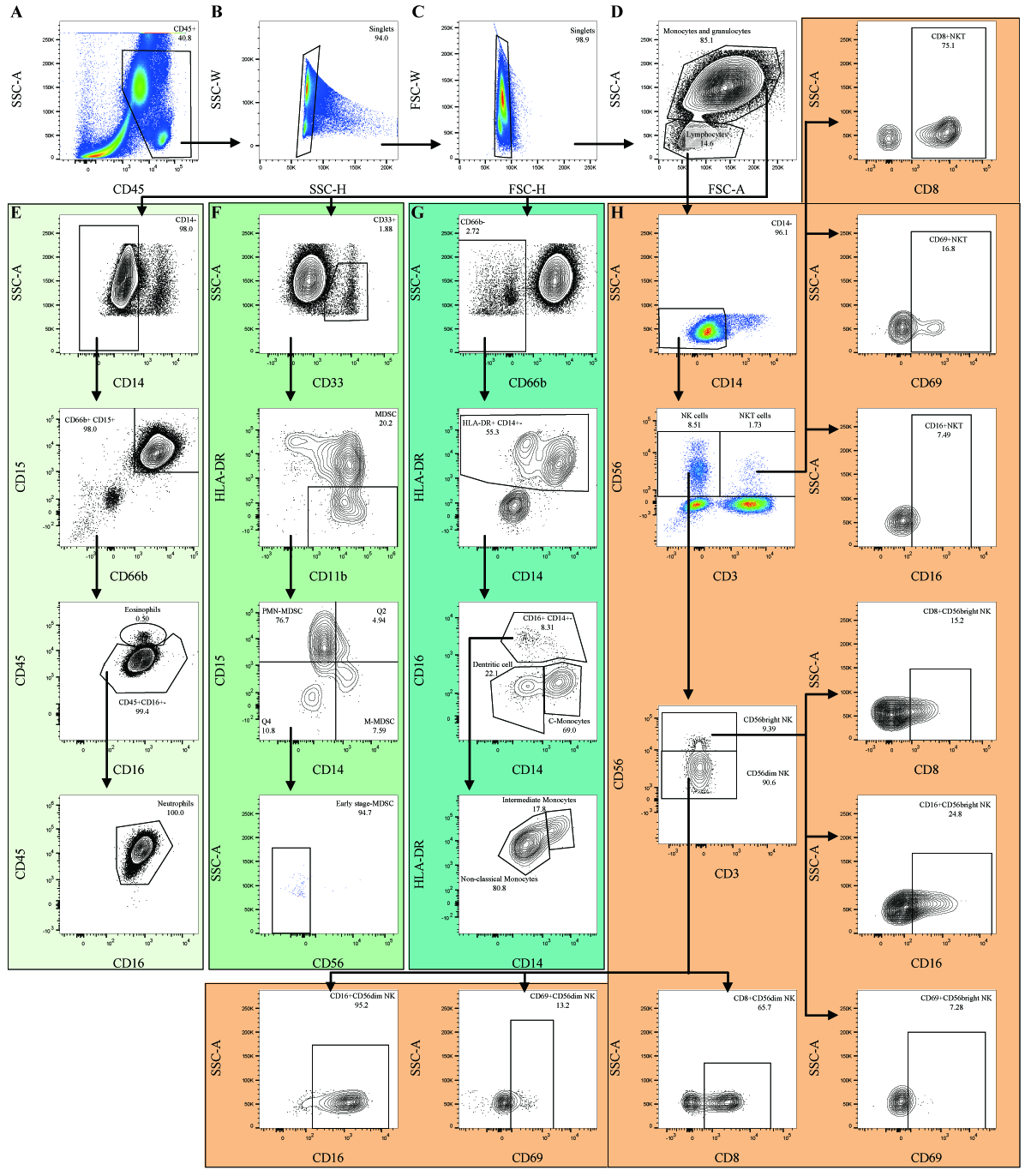

Supplement: Supplementary file 1 [file cancers-14-06105-s001.zip › Figure S3.tif]

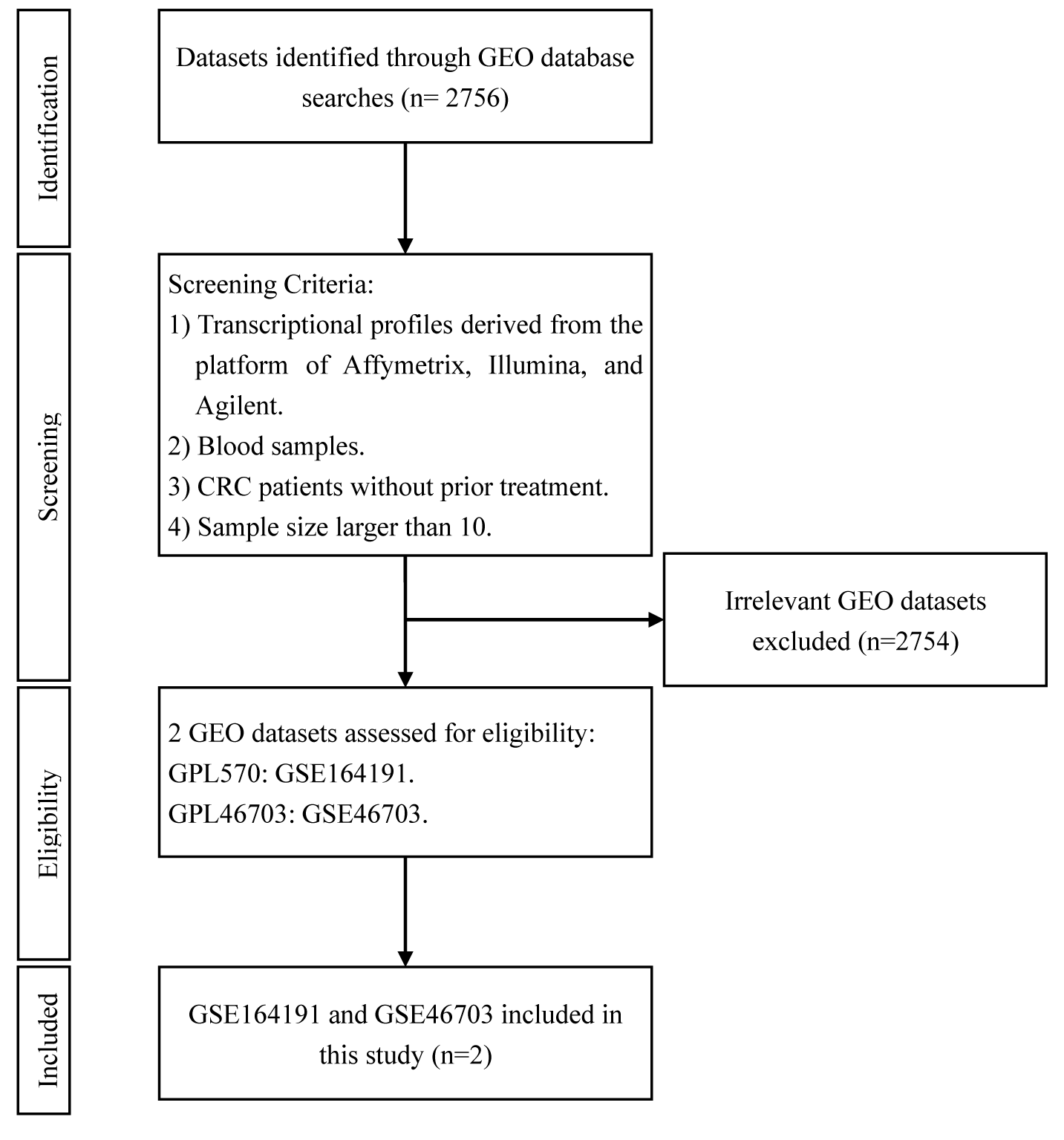

Supplement: Supplementary file 1 [file cancers-14-06105-s001.zip › Figure S4.tif]

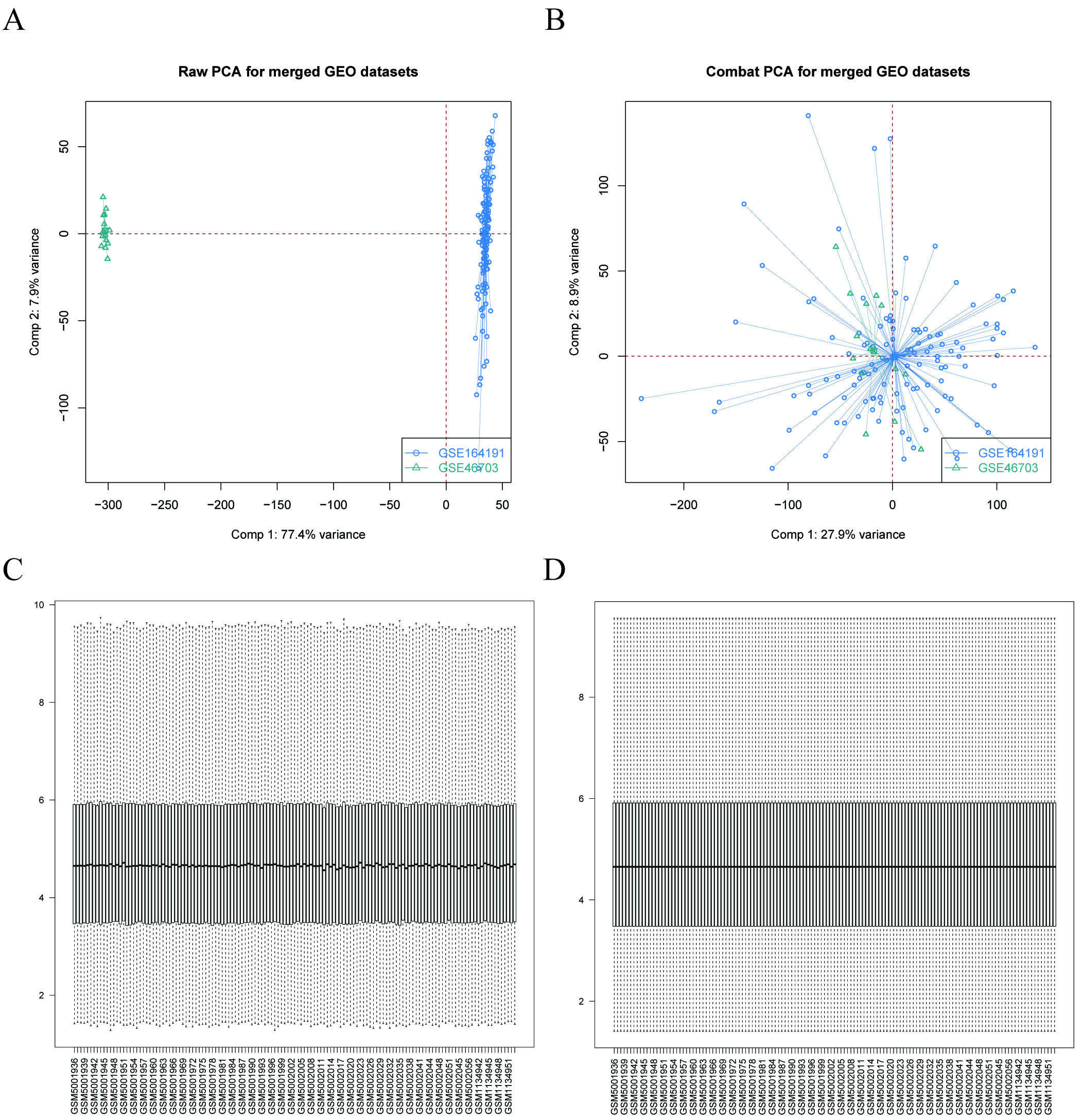

Supplement: Supplementary file 1 [file cancers-14-06105-s001.zip › Figure S5.tif]

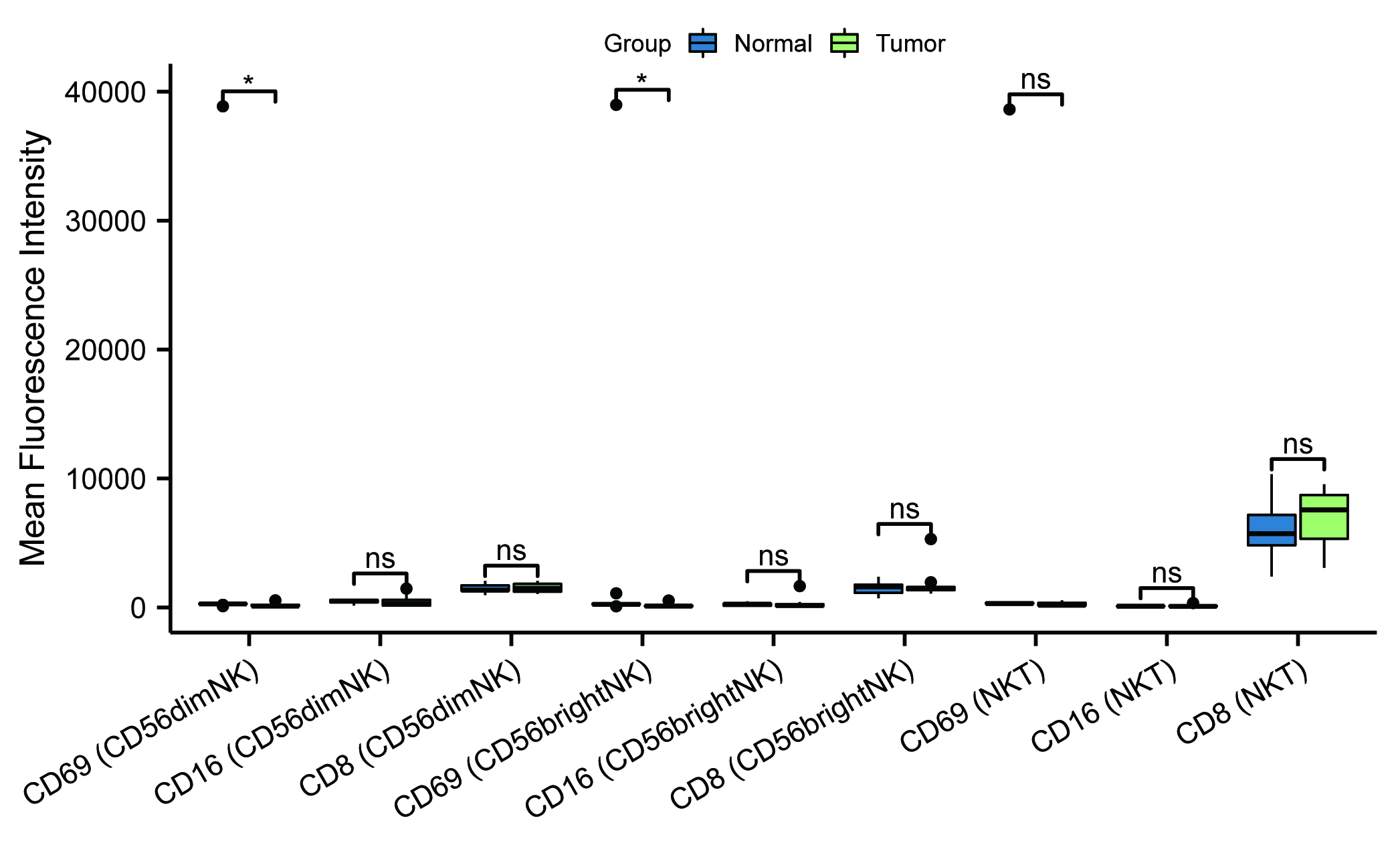

Supplement: Supplementary file 1 [file cancers-14-06105-s001.zip › Figure S6.tif]

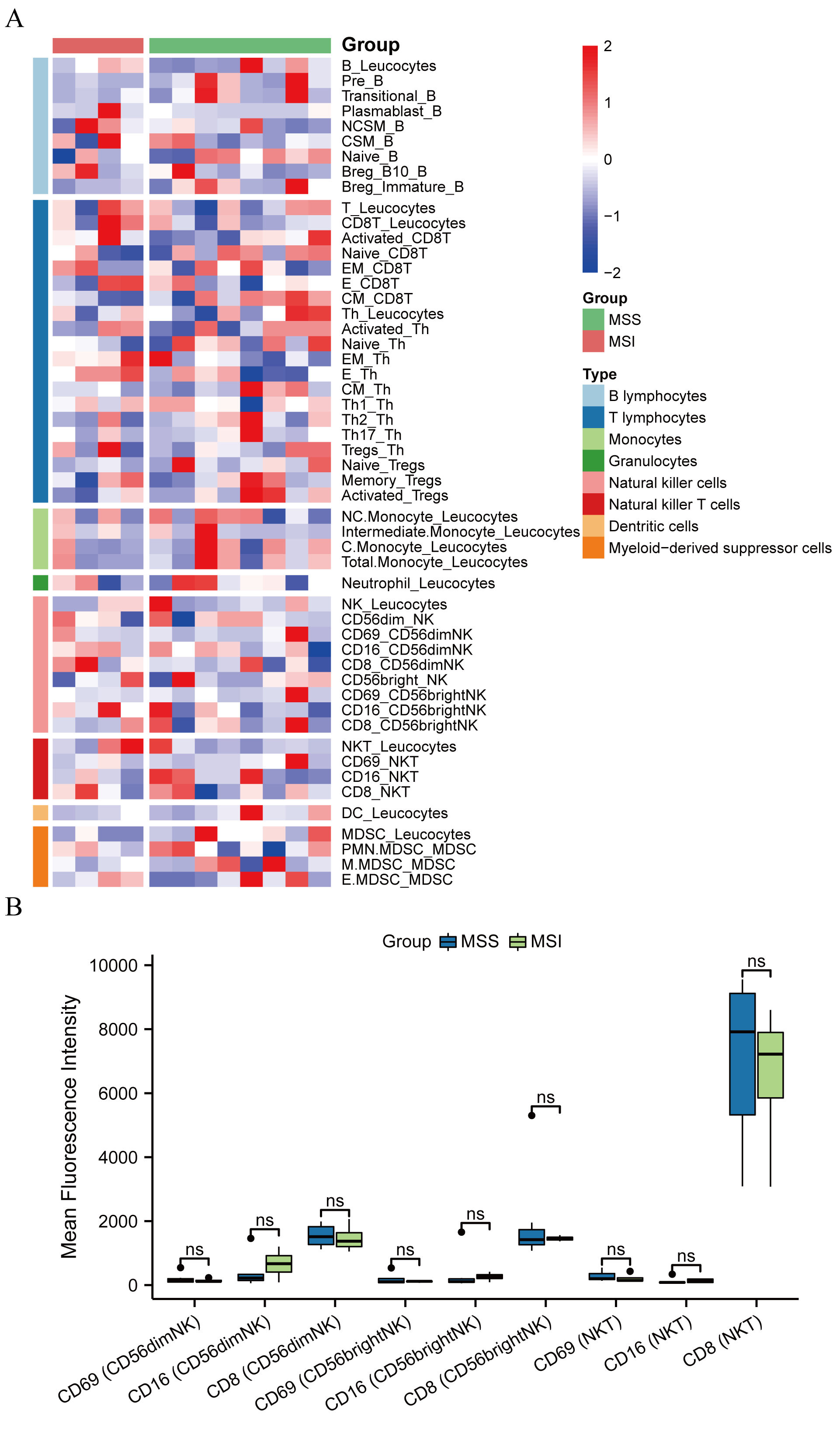

Supplement: Supplementary file 1 [file cancers-14-06105-s001.zip › Figure S7.tif]
